# LncRNA HOTAIR Inhibits miR-19a-3p to Alleviate Foam Cell Formation and Inflammatory Response in Atherosclerosis

**DOI:** 10.7150/ijms.90315

**Published:** 2024-01-12

**Authors:** Heming Chen, Xiaoyi Li, Weiqun Chen, Tangwei Wu, Shuiyi Liu

**Affiliations:** 1 Department of Cardiovascular Surgery, The Second XiangYa Hospital, Central South University, Changsha, Hunan, 410011, China.; 2 Department of Medical Laboratory, The Central Hospital of Wuhan, Wuhan, Hubei, 430014, China.; 3 Key Laboratory for Molecular Diagnosis of Hubei Province, The Central Hospital of Wuhan, Wuhan, Hubei, 430014, China.

**Keywords:** LncRNA, Atherosclerosis, Foam cell, Inflammatory factor

## Abstract

**Background:** Atherosclerosis, a chronic inflammatory disease, poses a significant risk for cardiovascular disorders. Meanwhile, emerging evidence suggests that long noncoding RNAs (lncRNAs) play pivotal roles in diverse cardiovascular conditions. Nonetheless, the functional implications of lncRNAs in atherosclerosis remain largely unexplored.

**Methods:** Quantitative real-time polymerase chain reaction (qRT-PCR) was employed to assess lncRNA HOTAIR and miR-19a-3p expression levels in patients with atherosclerosis and macrophage-derived foam cells. The release of inflammatory factors was evaluated using enzyme-linked immunosorbent assay (ELISA), while lipid uptake by foam cells was assessed through Oil Red O staining. Additionally, the targeting relationship between lncRNA HOTAIR and miR-19a-3p was validated via a Luciferase reporter assay.

**Results:** LncRNA HOTAIR exhibited downregulation in the plasma of atherosclerosis patients and was found to be inhibited by ox-LDL in human macrophage-derived foam cells. Overexpression of HOTAIR effectively reduced lipid uptake and suppressed the inflammatory response by downregulating the expression of TNF-α and IL-6 during foam cell formation. Mechanistically, HOTAIR mitigated foam cell formation by repressing the expression of miR-19a-3p.

**Conclusions:** In conclusion, our findings, in conjunction with previous studies, elucidate the role of HOTAIR in atherosclerosis. Specifically, we demonstrate that HOTAIR plays a role in alleviating foam cell formation and suppressing the inflammatory response by inhibiting miR-19a-3p in the context of atherosclerosis. Our results suggest the involvement of the TNF-α/miR-19a/HBP1/MIF pathway in mediating these effects. These findings contribute to a better understanding of atherosclerosis's molecular mechanisms and highlight the potential therapeutic implications of targeting HOTAIR and its associated pathways.

## 1. Introduction

Atherosclerosis, a pervasive pathological condition, underlies several critical threats to human health, including coronary heart disease, stroke, and peripheral vascular diseases^1^, ^2^. These conditions collectively account for most cardiovascular morbidity and mortality^3^. Extensive evidence suggests that chronic inflammation plays a crucial role in the development of atherosclerosis. Inflammatory factors and oxidative stress stimulate monocytes, gradually differentiating them into macrophages^4^. During this process, macrophages accumulate and ingest oxidized low-density lipoprotein (ox-LDL), forming lipid-overloaded foam cells. Foam cells play a significant role in the development of atherosclerotic plaques and the progression of atherosclerosis^5^-^7^.

Increasing evidence has substantiated the significant regulatory roles of noncoding RNAs in various biological processes^8^-^10^. Among these noncoding RNAs, long noncoding RNAs (lncRNAs), which exceed 200 nucleotides, have emerged as critical regulators that modulate gene expression and signaling pathways at different stages^11^-^13^. Atherosclerosis-associated lncRNAs have garnered considerable attention due to their regulatory effects on lipid metabolism, inflammatory responses, cell proliferation, apoptosis, adhesion, and migration^14^-^18^. One such lncRNA is HOX transcription antisense RNA (HOTAIR), transcribed from the antisense strand of the HOXC gene cluster and spanning 2.2 kilobases^19^. HOTAIR, one of the earliest identified lncRNAs, has been shown to be a vital regulator in the progression of various cancer types^20^-^22^. However, the precise mechanisms underlying the role of lncRNA HOTAIR in atherosclerosis remain largely unexplored, even though a previous study has reported downregulation of HOTAIR in atherosclerosis and its ability to alleviate the inflammatory response by promoting FXR1 expression in Raw264.7 cells has been demonstrated^23^. Additionally, HOTAIR has been reported to be downregulated, leading to the upregulation of SIRT1 through miR-34a sponge activity in diabetic cardiomyopathy^24^.

Nonetheless, the molecular mechanisms through which this lncRNA regulates the process of atherosclerosis remain largely unclear.

Our previous research elucidated the role of miR-19a-3p in atherosclerosis through the miR-19a/HBP1/MIF pathway upon that foundation, the current study aimed to investigate the expression levels of HOTAIR in both the plasma of atherosclerosis patients and foam cells. We observed that HOTAIR modulated lipid uptake and the inflammatory response during foam cell formation. Furthermore, our findings demonstrated that HOTAIR acted as a sponge for miR-19a-3p, resulting in the downregulation of HBP1 expression and the upregulation of MIF, consequently accelerating the secretion of inflammatory factors TNF-α and IL-6. Collectively, our study highlights HOTAIR as a promising therapeutic target for attenuating foam cell formation and mitigating the inflammatory response associated with atherosclerosis.

## 2. Materials and Methods

### 2.1 Patient characteristics and monocyte collection

A total of 100 blood samples (57 males, 43 females and the age of them were 64±12, 67±9) were prospectively collected from patients diagnosed with atherosclerotic coronary heart disease, confirmed through coronary angiography at the Central Hospital of Wuhan between January 2022 and June 2022. The diagnostic criteria for atherosclerosis were based on internal medicine guidelines and the latest diagnostic and treatment guidelines for coronary heart disease. Patients with infectious diseases, acute coronary syndrome, and acute myocardial infarction were excluded from the study. Concurrently, to get the appropriate and closely matched control group to better explore the expression between the patients and control, 100 healthy individuals (46 males, 54 females and the age of them were 63±12, 64±11) with normal results from physical examinations were selected. And there was no age difference between the patients and the normal controls (*P* value of male between two groups was 0.532 and female was 0.153, *P>0.05*). The inclusion criteria for the control group were as follows: 1) absence of hypertension, smoking, diabetes, hyperlipidemia, and other risk factors for coronary heart disease, 2) no acute infectious diseases such as upper respiratory tract infection, pneumonia, or bronchial asthma, with normal blood counts, 3) no history of cardiovascular genetic diseases, 4) absence of carotid artery wall plaque formation according to carotid ultrasound, and 5) normal blood lipid and other biochemical indicators. Monocytes were isolated using gradient centrifugation and stored at -80°C for subsequent analysis. This study protocol was approved by the Medical Ethics Committee of The Central Hospital of Wuhan, and informed consent was obtained from all participants before their enrollment.

### 2.2 Cell culture and establishing the foam cell model

HEK-293T cells and human monocytes THP-1 cells were procured from the American Type Culture Collection (ATCC). THP-1 cells in the logarithmic growth phase were seeded into 12-well cell culture plates at a concentration of 1×10^6^ cells/ml to induce foam cell formation. Each well was treated with 100 nM phorbol-12-myristate-13-acetate (PMA, Sigma-Aldrich) for 24 hours to promote the differentiation of adherent macrophages with pseudopodia. Subsequently, 50 μg/ml oxidized low-density lipoprotein (ox-LDL, Luwen) was added for 24 hours to induce the transformation of macrophages into foam cells^25^.

### 2.3 Cell transfection

The miR-19a-3p mimic, miR-19a-3p inhibitor, and their corresponding negative controls were obtained from RiboBio (Guangzhou, China). The full-length sequence of HOTAIR was cloned into a pcDNA-3.1 vector (Invitrogen) and confirmed by sequencing. Transfection was conducted using ExFect® Transfection reagent (Vazyme) following the manufacturer's instructions.

### 2.4 Enzyme-linked immunosorbent assay (ELISA)

The supernatant from THP-1 macrophages was collected, and the concentrations of secreted inflammatory factors TNF-α and IL-6 were measured using specific ELISA kits (R&D Systems) following the manufacturer's instructions.

### 2.5 Oil Red O staining

Macrophages derived from THP-1 cells were co-transfected with a miR-19a mimic or HOTAIR overexpression vector using ExFect® Transfection reagent (Vazyme). After 24 hours, the cells were treated with 50 μg/ml ox-LDL for an additional 24 hours to induce foam cell formation^25^-^27^. Subsequently, the foam cells were washed three times with 1× phosphate-buffered saline (PBS), fixed with 4% paraformaldehyde for 30 minutes, and stained with Oil Red O stain (Beisuo) for 20 minutes, following the manufacturer's instructions. Images of the foam cells were captured under a microscope (Olympus) at 400× magnification. The mean grey value of the Oil Red O staining was quantified using Image J software.

### 2.6 RNA isolation and qRT-PCR

Plasma miRNA from both healthy controls and patients with atherosclerosis (AS) was extracted using TRIzol reagent (Invitrogen). Total RNA from macrophages was also extracted using TRIzol reagent (Invitrogen). The extracted total RNA was reverse transcribed into cDNA and quantified using SYBR Green (Vazyme) on an ABI QuantStudio DX qPCR instrument. The primer sequences of HOTAIR were: Forward 5'-AGAGTTACAGACGGCGGCGA-3', Reverse 5'-TCTCTTCCCTCCTCTGGCTC-3'.

### 2.7 Luciferase reporter assay

The wild-type or mutant forms of HOTAIR were cloned into pRL-TK vectors, and the pGL3 vector (Promega) was used as a control. These constructs were co-transfected into HEK-293T cells with miR-19a-3p mimic or miR-19a-3p inhibitor using ExFect® Transfection reagent (Vazyme). After 48 hours of transfection, the luciferase activity was measured using the Dual-Glo luciferase reporter assay system (Promega). The luciferase activity values were quantified using a Victor X2 multilabel reader (PerkinElmer).

### 2.8 Statistical analysis

The data from the two groups were compared using Student's t-test, while the data among multiple groups were analyzed using one-way analysis of variance (ANOVA) with the aid of SPSS 17.0 software. Pearson analysis was used for the analysis of correlation between HOTAIR and miR-19a-3p expression. Three independent measurements were conducted throughout the study. A p-value less than 0.05 was considered statistically significant. The significance levels were denoted as follows: * for p<0.05, ** for p<0.01, and *** for p<0.001.

## 3. Results

### 3.1 HOTAIR is downregulated in the plasma of patients with atherosclerosis and inhibited by ox-LDL in human macrophage-derived foam cells

**Figure 1:**
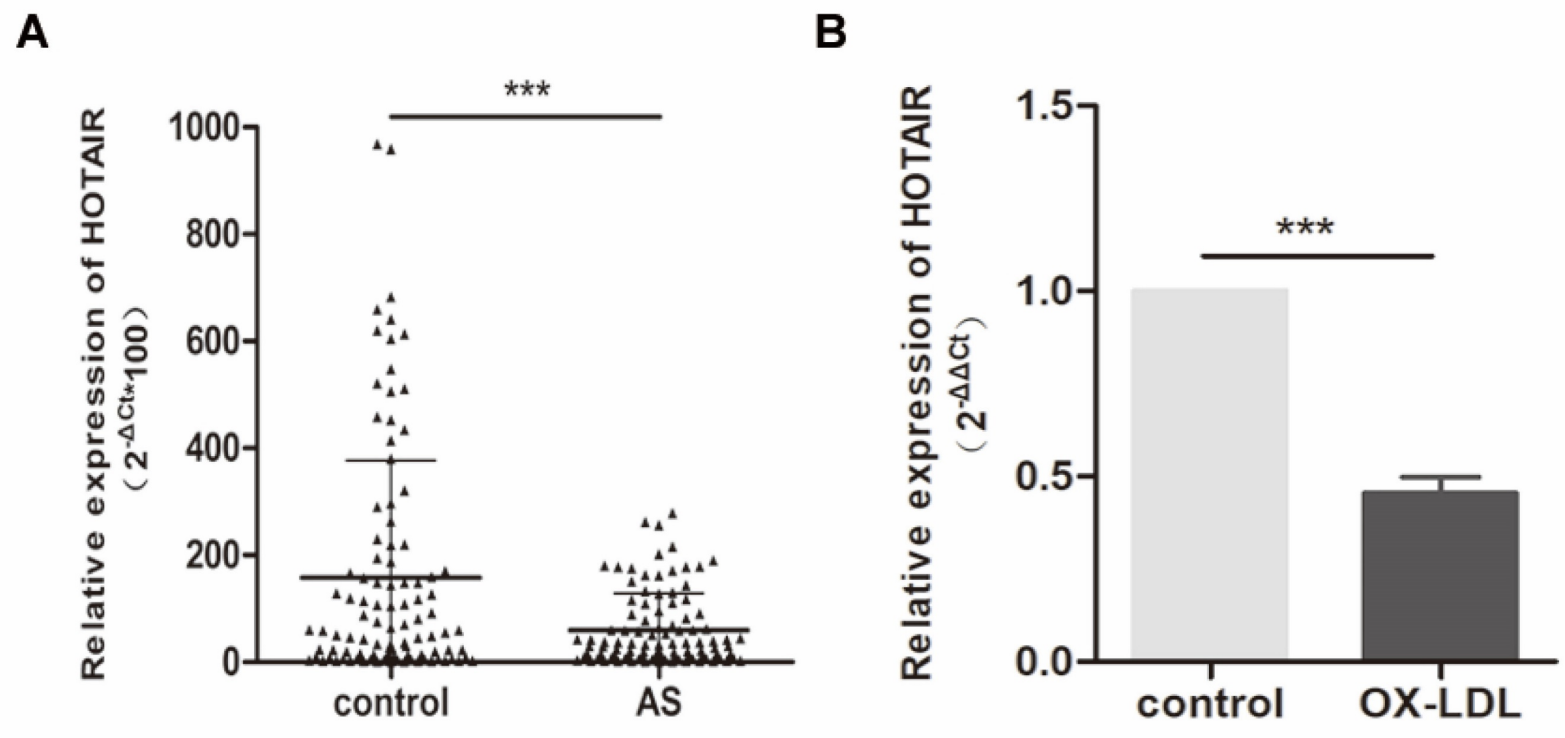
**Expression of HOTAIR in Plasma of Atherosclerosis Patients and Macrophage-Derived Foam Cells. A**: qRT-PCR analysis showing the expression levels of HOTAIR in plasma samples from 100 atherosclerosis patients compared to 100 healthy controls. **B**: qRT-PCR analysis demonstrating the expression levels of HOTAIR in macrophage-derived foam cells and control cells. The expression levels were normalized to appropriate reference genes, and statistical significance was determined using Student's t-test (*P<0.05, **P<0.01, ***P<0.001). Error bars represent standard deviation.

To investigate the expression of HOTAIR in individuals with atherosclerosis, we assessed its levels in plasma samples obtained from 100 atherosclerosis patients and 100 healthy controls. Our analysis revealed a significant downregulation of HOTAIR in patients with atherosclerosis (Fig. 1A). Furthermore, we sought to examine HOTAIR expression in an in vitro foam cell model using THP-1 cells. Following treatment with 100 nM PMA and subsequent stimulation with oxLDL (50 μg/ml) for 24 hours, we observed a notable downregulation of HOTAIR expression (Fig. 1B). These findings provide evidence that HOTAIR is downregulated in the plasma of individuals with atherosclerosis and is further suppressed by oxLDL in human macrophage-derived foam cells.

### 3.2 Overexpression of HOTAIR mitigated lipid uptake and attenuated the inflammatory response by suppressing the expression of TNF-α and IL-6 during foam cell formation

**Figure 2:**
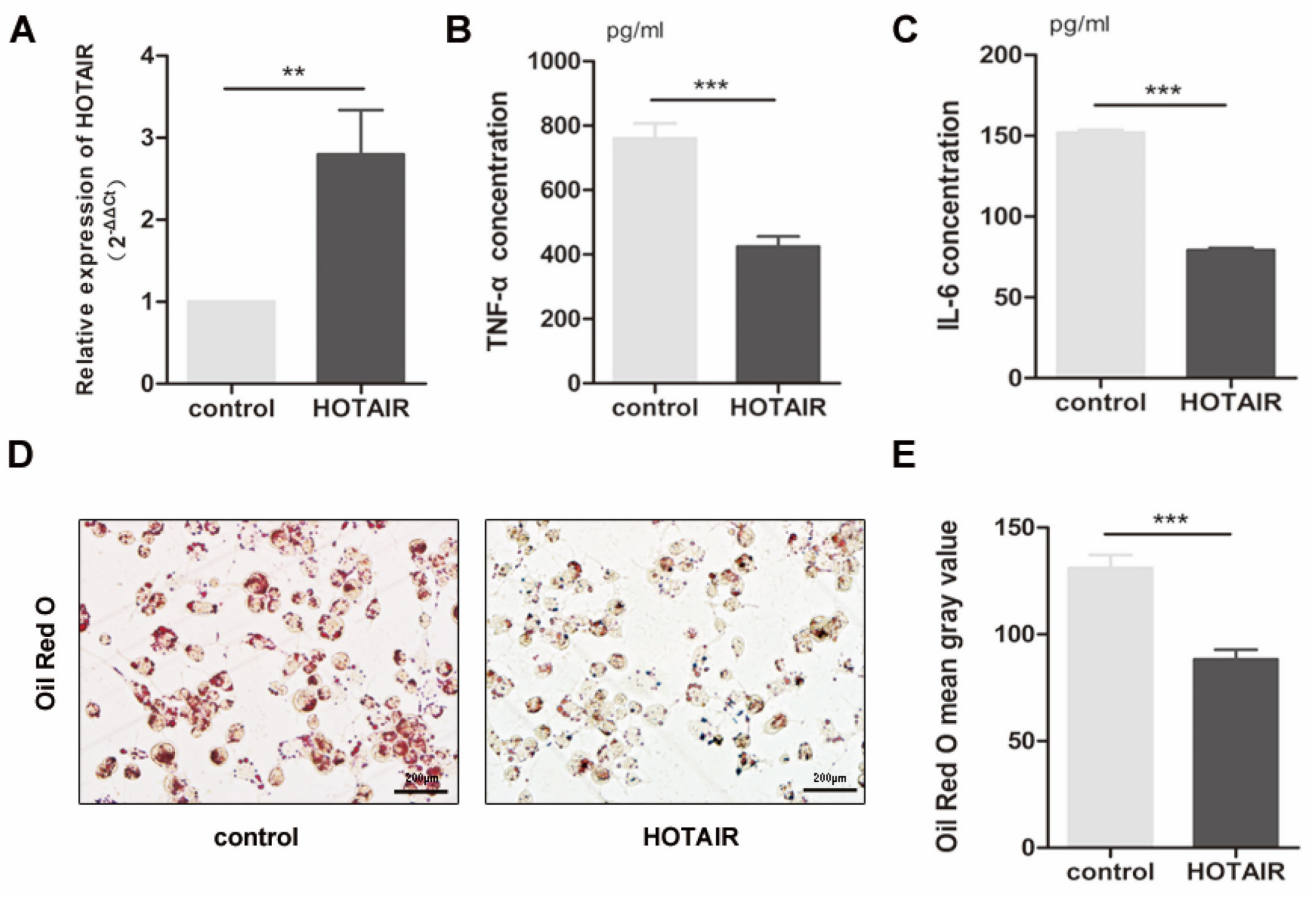
**Effects of HOTAIR overexpression on lipid uptake and inflammatory response in macrophage-derived foam cells. A**: qRT-PCR analysis of HOTAIR expression in macrophage-derived foam cells transfected with HOTAIR overexpression vector or control vector. **B** and **C**: ELISA measurements of TNF-α and IL-6 production in macrophage-derived foam cells transfected with HOTAIR overexpression vector or control vector. **D** and **E**: Oil Red O staining showing lipid uptake in macrophage-derived foam cells transfected with HOTAIR overexpression vector or control vector. Note: The expression levels were normalized to appropriate reference genes, and statistical significance was determined using Student's t-test (*P<0.05, **P<0.01, ***P<0.001). Error bars represent standard deviation.

In forming foam cells, the production of inflammatory cytokines and lipid uptake by macrophages play a crucial role. To investigate the impact of HOTAIR on the inflammatory response in atherosclerosis, we transfected macrophages, which were stimulated by PMA-treated THP-1 cells, with a HOTAIR overexpression plasmid for 24 hours, followed by treatment with ox-LDL for an additional 24 hours. We assessed the transfection efficiency of the HOTAIR overexpression using qRT-PCR (Fig. 2A). Furthermore, we analyzed the effect of HOTAIR overexpression on foam cell formation through Oil Red O staining and measured the secretion of inflammatory factors in the cell supernatant using ELISA. Our results demonstrated that HOTAIR overexpression in macrophages led to a suppression of IL-6 and TNF-α production (Fig. 2B, C) and a reduction in lipid uptake (Fig. 2D, E).

### 3.3 HOTAIR downregulated the expression of miR-19a-3p

**Figure 3:**
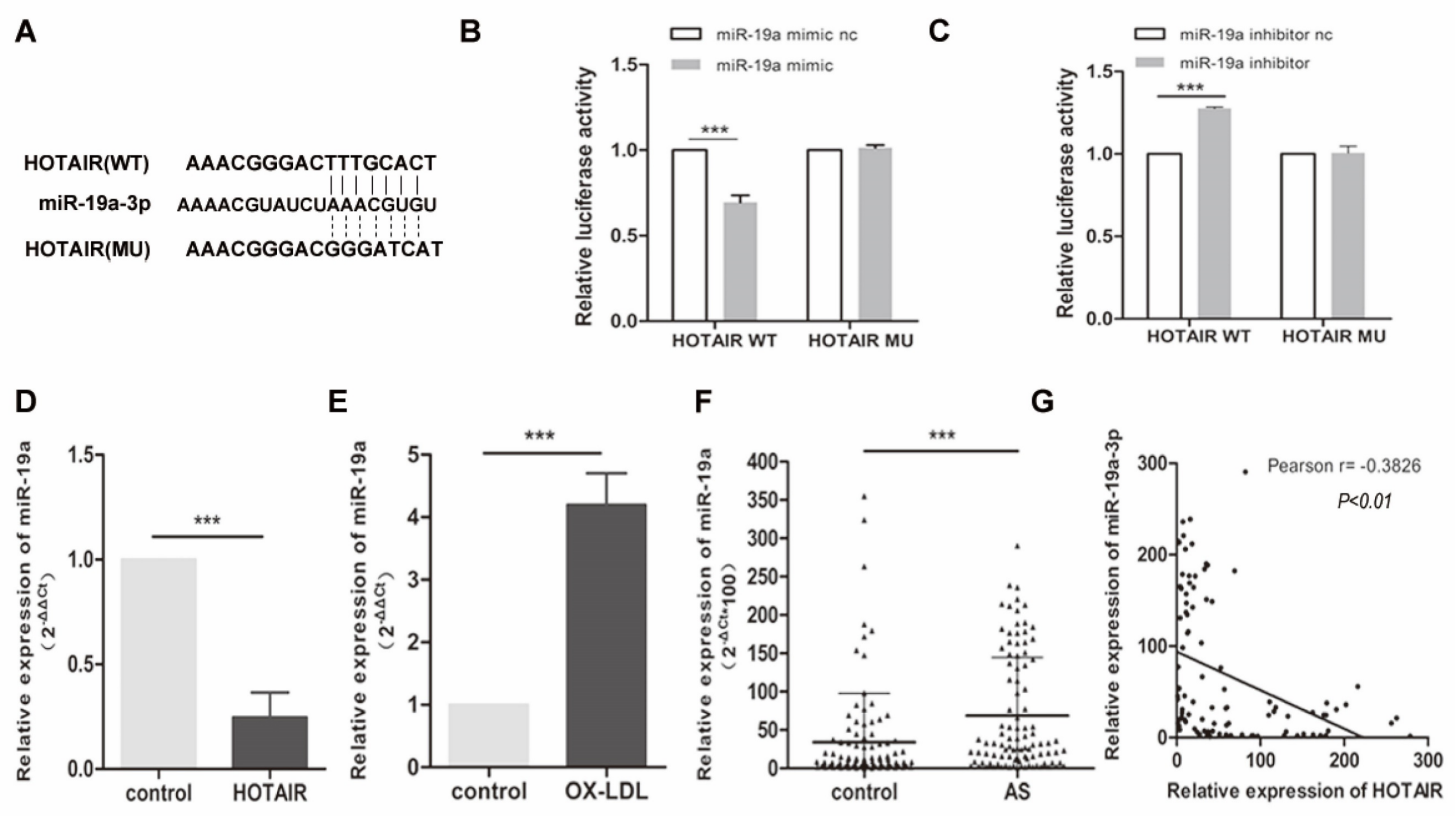
**Relationship between HOTAIR and miR-19a-3p and Expression of miR-19a-3p in Macrophage-Derived Foam Cells. A**: Predicted binding sites of HOTAIR and miR-19a-3p from StarBase V3.0, with indicated mutated binding sites. **B** and **C**: Luciferase reporter assay in HEK-293T cells co-transfected with pRL-TK carrying HOTAIR wildtype or mutant binding site and the miR-19a-3p mimic or the miR-19a-3p inhibitor. Luciferase activity was measured 48 hours post-transfection. **D**: qRT-PCR analysis of miR-19a-3p expression in macrophage-derived foam cells transfected with HOTAIR overexpression vector or control vector. **E**: qRT-PCR analysis of miR-19a-3p expression in macrophage-derived foam cells and control cells. **F**: qRT-PCR analysis of miR-19a-3p expression in plasma samples from 100 atherosclerosis patients compared to 100 healthy controls. **G**: Pearson analysis showing the correlation between HOTAIR and miR-19a-3p expression. Note: Statistical significance was determined using Student's t-test (*P<0.05, **P<0.01, ***P<0.001). Error bars represent standard deviation.

To unravel the mechanism underlying the impact of HOTAIR on macrophage-derived foam cell formation and the inflammatory response, we investigated potential miRNAs that interact with HOTAIR. Through analysis in StarBase V3.0, we identified miR-19a-3p as a potential binding partner for HOTAIR (Fig. 3A). To validate this interaction, we performed a luciferase reporter assay in HEK-293T cells. The results indicated that miR-19a overexpression significantly suppressed luciferase activity in the HOTAIR wildtype (WT) construct but not in the mutant (MU) construct (Fig. 3B). Moreover, downregulation of miR-19a led to a notable increase in luciferase activity specifically with the WT HOTAIR construct, but not the MU construct (Fig. 3C).

To further confirm the relationship between HOTAIR and miR-19a, we measured the expression of miR-19a-3p in ox-LDL-treated macrophages with or without HOTAIR overexpression using qRT-PCR. The results demonstrated that HOTAIR overexpression in macrophage-derived foam cells led to a downregulation of miR-19a-3p expression (Fig. 3D). Additionally, the expression of miR-19a-3p was found to be higher in macrophage-derived foam cells compared to control cells (Fig. 3E). Furthermore, we examined the levels of miR-19a-3p in plasma samples obtained from 100 atherosclerosis patients and healthy controls. The data revealed that miR-19a-3p levels were elevated in the plasma of atherosclerosis patients compared to healthy controls (Fig. 3F). Notably, there was a negative correlation between the expression of HOTAIR and miR-19a-3p in the plasma of the 100 atherosclerosis patients (Fig. 3G).

### 3.4 HOTAIR suppresses mir-19a-3p expression to alleviate lipid uptake and inhibit inflammatory reactions in foam cell formation

**Figure 4:**
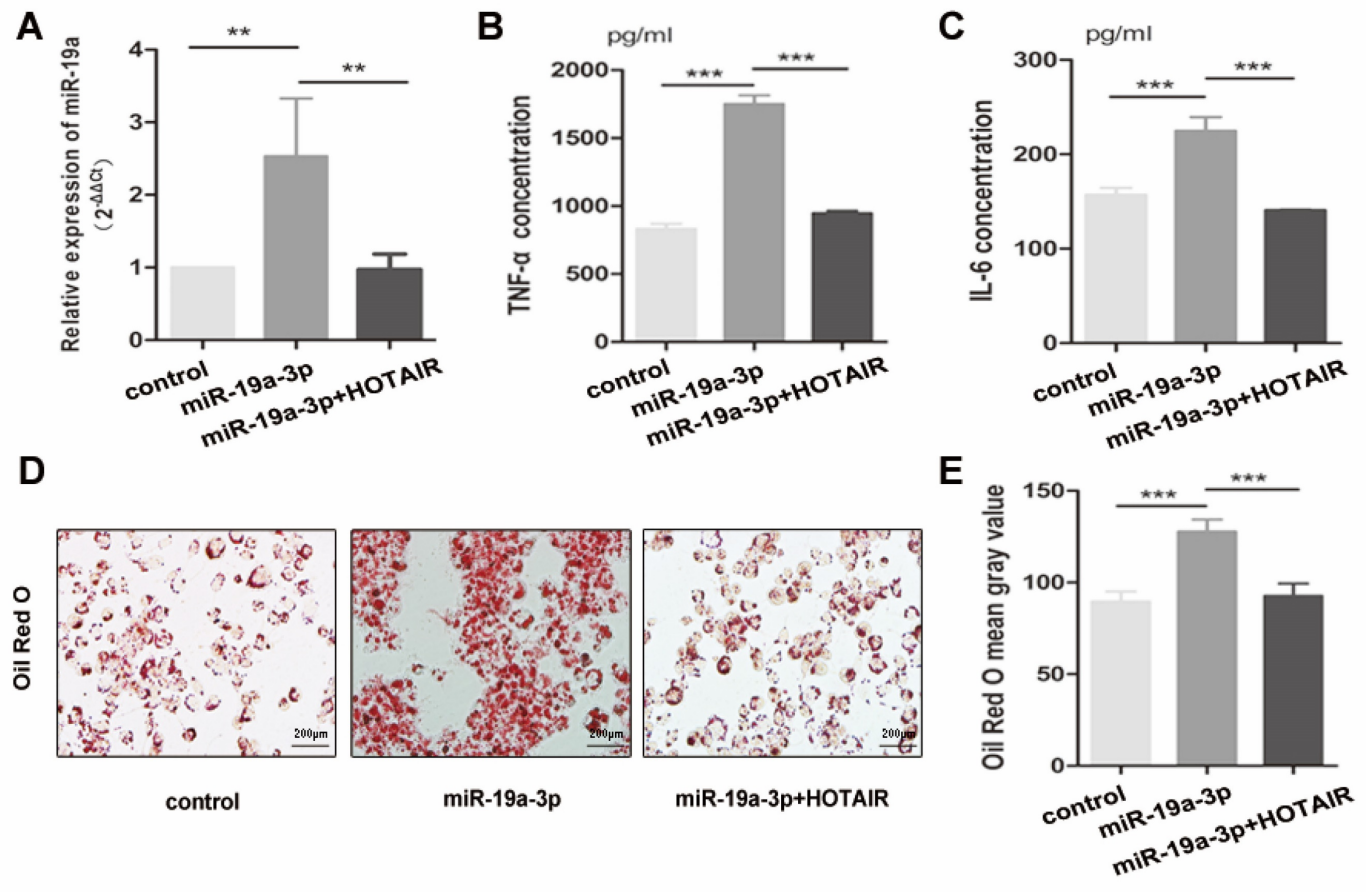
** HOTAIR alleviates lipid uptake and suppresses the inflammatory response by repressing the expression of mir-19a-3p in foam cell formation. A**: qRT-PCR analysis of HOTAIR expression in macrophage-derived foam cells transfected with HOTAIR overexpression vector and/or miR-19a-3p mimic. **B** and **C**: ELISA assay for TNF-α and IL-6 production in macrophage-derived foam cells transfected with HOTAIR overexpression vector and/or miR-19a-3p mimic. **D** and **E**: Oil Red O staining for lipid uptake in macrophage-derived foam cells transfected with HOTAIR overexpression vector and/or miR-19a-3p mimic. Note: Statistical significance was determined using Student's t-test (*P<0.05, **P<0.01, ***P<0.001). Error bars represent standard deviation.

To further investigate the impact of HOTAIR on lipid uptake and inflammatory response in foam cell formation, we conducted transfection experiments in macrophage-derived foam cells using the HOTAIR overexpression vector in combination with the miR-19a-3p mimic. The introduction of the HOTAIR overexpression vector resulted in a decrease in miR-19a-3p expression in THP-1-derived macrophages (Fig. 4A). The elevated protein levels of TNF-α and IL-6 induced by miR-19a-3p overexpression were reversed by HOTAIR overexpression in macrophage-derived foam cells (Fig. 4B, C). Furthermore, Oil Red O staining results indicated that the increased lipid uptake mediated by miR-19a-3p overexpression was counteracted by co-transfection with the HOTAIR overexpression vector (Fig. 4D-4E). These findings demonstrate that HOTAIR mitigates lipid uptake and inhibits the inflammatory reaction by suppressing the expression of miR-19a-3p during foam cell formation.

## 4. Discussion

Despite significant advancements in diagnostics and therapeutics in recent decades, cardiovascular diseases (CVDs) remain the leading global cause of mortality^28^. Among CVDs, angina, myocardial infarction, and ischemic stroke contribute significantly to morbidity and mortality worldwide^29^. Atherosclerosis has been identified as a significant underlying cause of CVDs^30^. Atherosclerosis is a progressive condition characterized by excessive lipid accumulation in the artery walls (intima) and chronic secretion of inflammatory factors within the blood vessels^31^, ^32^. Both inflammatory response and oxidative stress are regarded as two critical contributors to atherosclerosis^33^, and macrophages play an important role in the pathogenesis of atherosclerosis by mediating oxidative stress, inflammation and lipid metabolism, which can lead to the formation of vascular plaque^34^, ox-LDL-induced oxidative stress via changing the levels of MDA, ROS and SOD. In our research, wo focused on the role of inflammation and lipid metabolism by THP-1-derived macrophages^35^, ^36^. In the development of atherosclerosis, macrophages within the atherosclerotic plaque take up modified lipoproteins and undergo transformation into foam cells through stimulation by inflammatory factors. These processes are considered critical and initial steps in the pathogenesis of atherosclerosis. Although extensive research has been conducted on the pathological mechanisms of atherosclerosis, given its genetic susceptibility nature, exploring the transcriptome level can provide a comprehensive understanding of the disease's pathogenesis and offer the potential for identifying new biomarkers and therapeutic targets. Therefore, investigating the molecular mechanisms and pathogenesis of atherosclerosis holds immense significance.

LncRNAs play diverse roles in gene expression and signaling pathways at various stages, functioning as decoys, guides, signaling mediators, or scaffolds^37^. They also interact with miRNAs to regulate the target genes of these miRNAs. Mounting evidence suggests that lncRNA dysregulation is closely associated with human diseases, including atherosclerosis, where they play significant roles. For instance, the lncRNAs H19, TUG1, and Neat1 are upregulated in atherosclerosis and contribute to vascular smooth muscle cell (VSMC) proliferation while inhibiting apoptosis On the other hand, the downregulation of UCA1, MEG3, lincRNA-p21, and GASL1 in atherosclerosis leads to suppressed VSMC proliferation and inflammation^8^, ^38^-^40^. In our previous research, we observed increased expression of miR-19a-3p in the serum and atherosclerotic plaques of patients with atherosclerosis. We found that miR-19a-3p suppresses HBP1 expression and upregulates MIF, resulting in the release of inflammatory factors TNF-α and IL-6. This pro-inflammatory and pro-atherogenic pathway involving TNF-α, miR-19a-3p, HBP1, and MIF forms a positive feedback loop^41^. Given the known interaction between lncRNAs and miRNAs in regulating target genes, we hypothesize that the increased expression of miR-19a-3p in the serum and atherosclerotic plaques of patients with atherosclerotic coronary heart disease may be regulated by specific lncRNAs.

HOTAIR is a lncRNA that spans 2158 base pairs and is located on the antisense strand of the HOXC gene locus on chromosome 12, between HOXC11 and HOXC12. It has been extensively studied in various tumor progression contexts and is associated with tumor size, advanced metastasis, and extensive metastasis. More recently, reports have suggested that HOTAIR may also play a role in cardiovascular diseases. For instance, in Chronic Obstructive Pulmonary Disease (COPD), HOTAIR has been found to facilitate pulmonary vascular endothelial cell apoptosis^42^. In diabetic cardiomyopathy, HOTAIR acts as a competing endogenous RNA (ceRNA) to increase SIRT1 expression by sponging miR-34a^24^. Additionally, in obesity-induced myocardial injury, HOTAIR regulates the release of ghrelin^43^. However, the specific role of HOTAIR in the pathogenesis of atherosclerosis has yet to be determined. To address this knowledge gap, our study focused on examining the expression of HOTAIR in plasma samples obtained from 100 patients with atherosclerosis and healthy controls. Our findings revealed that HOTAIR expression was downregulated in patients with atherosclerosis. Furthermore, we investigated the impact of ox-LDL (oxidized low-density lipoprotein) on HOTAIR expression in human macrophages and observed its inhibitory effect. Building upon these observations, we explored the potential mechanisms underlying HOTAIR's effects on foam cell formation and macrophage inflammatory response. To identify potential interacting miRNAs, we searched the StarBase V3.0 database, which predicted an interaction between HOTAIR and miR-19a-3p. Subsequent experiments revealed that HOTAIR overexpression attenuated lipid uptake and suppressed the production of inflammatory factors IL-6 and TNF-α in macrophages. These effects were attributed to the repression of miR-19a-3p expression by HOTAIR in the context of foam cell formation. Overall, our study provides novel insights into the regulatory role of HOTAIR in atherosclerosis and highlights its potential as a therapeutic target for modulating lipid uptake and inflammatory responses in foam cell formation.

**Figure 5:**
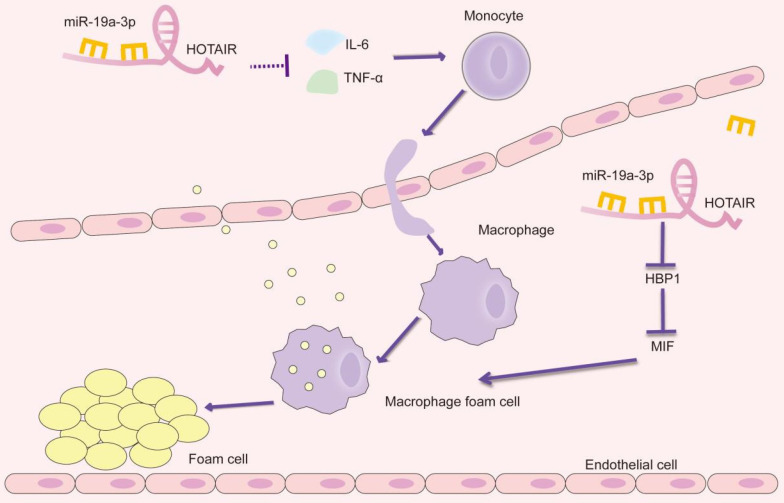
Schematic presentation of the HOTAIR/miR-19a-3p/HBP1/MIF axis in the foam cell formation and inflammatory response in atherosclerosis.

By integrating our current findings with previous studies, we have uncovered the role of HOTAIR in atherosclerosis. Our results suggest that HOTAIR plays a significant part in alleviating foam cell formation and reducing the inflammatory response in the context of atherosclerosis. This effect is achieved through the inhibition of miR-19a-3p, which involves the miR-19a-HBP1-MIF pathway (Figure 5). Our findings provide valuable insights into the molecular mechanisms underlying the involvement of HOTAIR in atherosclerosis. By targeting the miR-19a-3p-HBP1-MIF pathway, HOTAIR represents a potential therapeutic target for modulating the inflammatory response and foam cell formation, offering promising avenues for the development of novel treatments for atherosclerosis. Identifying HOTAIR as a critical regulator in atherosclerosis further highlights the importance of lncRNAs in CVDs. It expands our understanding of the complex molecular networks involved in the pathogenesis of atherosclerosis.

## 5. Conclusion

Combined with present and previous studies, we uncovered the involvement of HOTAIR in atherosclerosis, at least in part, we demonstrate that HOTAIR alleviates the formation of foam cell and inflammatory reaction by inhibiting miR-19a-3p in atherosclerosis through TNF-α/miR-19a/HBP1/MIF pathway.

These findings contribute to a better understanding of atherosclerosis's molecular mechanisms and highlight the potential therapeutic implications of targeting HOTAIR and its associated pathways.

## Abbreviations

AS: Atherosclerosis
LncRNAs: Long noncoding RNAs
QRT-PCR: Quantitative real-time polymerase chain reaction
HOTAIR: HOX transcription antisense RNA
MiRNAs: MicroRNAs
ELISA: Enzyme-linked immunosorbent assay
Ox-LDL: Oxidized low-density lipoprotein
TNF-α: Tumor necrosis factor-α
IL-6: Interleukin-6
HBP1: HMG-Box Transcription Factor 1
MIF: Macrophage migration inhibiting factor
HEK-293T: Human embryonic kidney 293T
THP-1: Human myeloid leukemia mononuclear cells
ATCC: American Type Culture Collection
PMA: Phorbol 12-myristate 13-acetate
ANOVA: One-way analysis of variance
MDA: Malondialdehyde
ROS: Reactive oxygen species
SOD: Superoxide dismutase

## References

[1] Tsao CW, Aday AW, Almarzooq ZI (2023). Heart Disease and Stroke Statistics-2023 Update: A Report From the American Heart Association. Circulation.

[2] Libby P, Buring JE, Badimon L (2019). Atherosclerosis. Nat Rev Dis Primers.

[3] Shelbaya K, Claggett B, Dorbala P (2023). Stages of Valvular Heart Disease Among Older Adults in the Community: The Atherosclerosis Risk in Communities Study. Circulation.

[4] Hansson GK (2005). Inflammation, atherosclerosis, and coronary artery disease. N Engl J Med.

[5] Fernández-Ruiz I (2022). Macropinocytosis promotes foam cell formation and atherosclerosis. Nat Rev Cardiol.

[6] Edsfeldt A, Swart M, Singh P (2022). Interferon regulatory factor-5-dependent CD11c+ macrophages contribute to the formation of rupture-prone atherosclerotic plaques. Eur Heart J.

[7] Li C, Qu L, Matz AJ (2022). AtheroSpectrum Reveals Novel Macrophage Foam Cell Gene Signatures Associated With Atherosclerotic Cardiovascular Disease Risk. Circulation.

[8] Wu G, Cai J, Han Y (2014). LincRNA-p21 regulates neointima formation, vascular smooth muscle cell proliferation, apoptosis, and atherosclerosis by enhancing p53 activity. Circulation.

[9] Ahmed ASI, Dong K, Liu J (2018). Long noncoding RNA NEAT1 (nuclear paraspeckle assembly transcript 1) is critical for phenotypic switching of vascular smooth muscle cells. Proc Natl Acad Sci U S A.

[10] Jourdi G, Godier A, Lordkipanidzé M (2022). Antiplatelet Therapy for Atherothrombotic Disease in 2022-From Population to Patient-Centered Approaches. Front Cardiovasc Med.

[11] Haemmig S, Yang D, Sun X (2020). Long noncoding RNA SNHG12 integrates a DNA-PK-mediated DNA damage response and vascular senescence. Sci Transl Med.

[12] Hu YW, Guo FX, Xu YJ (2019). Long noncoding RNA NEXN-AS1 mitigates atherosclerosis by regulating the actin-binding protein NEXN. J Clin Invest.

[13] Sallam T, Sandhu J, Tontonoz P (2018). Long Noncoding RNA Discovery in Cardiovascular Disease: Decoding Form to Function. Circ Res.

[14] Dai X, Liu S, Cheng L (2022). Epigenetic Upregulation of H19 and AMPK Inhibition Concurrently Contribute to S-Adenosylhomocysteine Hydrolase Deficiency-Promoted Atherosclerotic Calcification. Circ Res.

[15] Simion V, Zhou H, Haemmig S (2020). A macrophage-specific lncRNA regulates apoptosis and atherosclerosis by tethering HuR in the nucleus. Nat Commun.

[16] Ryu J, Ahn Y, Kook H (2021). The roles of non-coding RNAs in vascular calcification and opportunities as therapeutic targets. Pharmacol Ther.

[17] Zhang Y, Du W, Yang B (2019). Long non-coding RNAs as new regulators of cardiac electrophysiology and arrhythmias: Molecular mechanisms, therapeutic implications and challenges. Pharmacol Ther.

[18] Guo FX, Wu Q, Li P (2019). The role of the LncRNA-FA2H-2-MLKL pathway in atherosclerosis by regulation of autophagy flux and inflammation through mTOR-dependent signaling. Cell Death Differ.

[19] Yang L, Peng X, Li Y (2019). Long non-coding RNA HOTAIR promotes exosome secretion by regulating RAB35 and SNAP23 in hepatocellular carcinoma. Mol Cancer.

[20] Raju GSR, Pavitra E, Bandaru SS (2023). HOTAIR: a potential metastatic, drug-resistant and prognostic regulator of breast cancer. Mol Cancer.

[21] Chen S, Shen X (2020). Long noncoding RNAs: functions and mechanisms in colon cancer. Mol Cancer.

[22] Serghiou S, Kyriakopoulou A, Ioannidis JP (2016). Long noncoding RNAs as novel predictors of survival in human cancer: a systematic review and meta-analysis. Mol Cancer.

[23] Pang JL, Wang JW, Hu PY (2018). HOTAIR alleviates ox-LDL-induced inflammatory response in Raw264.7 cells via inhibiting NF-κB pathway. Eur Rev Med Pharmacol Sci.

[24] Gao L, Wang X, Guo S (2019). LncRNA HOTAIR functions as a competing endogenous RNA to upregulate SIRT1 by sponging miR-34a in diabetic cardiomyopathy. J Cell Physiol.

[25] Li X, Kong D, Chen H (2016). miR-155 acts as an anti-inflammatory factor in atherosclerosis-associated foam cell formation by repressing calcium-regulated heat stable protein 1. Sci. Rep.

[26] Chen H, Li X, Liu S (2017). MircroRNA-19a promotes vascular inflammation and foam cell formation by targeting HBP-1 in atherogenesis. Sci. Rep.

[27] Zhu G, Yang L, Guo R (2013). miR-155 inhibits oxidized low-density lipoprotein-induced apoptosis of RAW264.7 cells. Mol. Cell. Biochem.

[28] Qu K, Yan F, Qin X (2022). Mitochondrial dysfunction in vascular endothelial cells and its role in atherosclerosis. Front Physiol.

[29] Khan A (2023). Are we ready for cell-specific therapies in atherosclerosis?. Eur. Heart J.

[30] Ding J, Li H, Liu W (2022). miR-186-5p Dysregulation in Serum Exosomes from Patients with AMI Aggravates Atherosclerosis via Targeting LOX-1. Int J Nanomedicine.

[31] González L, Rivera K, Andia ME (2022). The IL-1 Family and Its Role in Atherosclerosis. Int J Mol Sci.

[32] Wang B, Tang X, Yao L (2022). Disruption of USP9X in macrophages promotes foam cell formation and atherosclerosis. J Clin Invest.

[33] Xu C, Chen L, Wang R (2022). LncRNA KCNQ1OT1 knockdown inhibits ox-LDL-induced inflammatory response and oxidative stress in THP-1 macrophages through the miR-137/TNFAIP1 axis. Cytokine.

[34] Xu L, Ren H, Xie D (2023). Rac2 mediate foam cell formation and associated immune responses in THP-1 to promote the process of atherosclerotic plaques. Mol. Immunol.

[35] Peng W, Li S, Chen S (2021). Hsa_circ_0003204 Knockdown Weakens Ox-LDL-Induced Cell Injury by Regulating miR-188-3p/TRPC6 Axis in Human Carotid Artery Endothelial Cells and THP-1 Cells. Front Cardiovasc Med.

[36] Liao Y, Zhu E, Zhou W (2021). Ox-LDL Aggravates the Oxidative Stress and Inflammatory Responses of THP-1 Macrophages by Reducing the Inhibition Effect of miR-491-5p on MMP-9. Front Cardiovasc Med.

[37] Uchida S, Dimmeler S (2015). Long noncoding RNAs in cardiovascular diseases. Circ Res.

[38] Tian S, Yuan Y, Li Z (2018). LncRNA UCA1 sponges miR-26a to regulate the migration and proliferation of vascular smooth muscle cells. Gene.

[39] Bai Y, Zhang Q, Su Y (2019). Modulation of the Proliferation/Apoptosis Balance of Vascular Smooth Muscle Cells in Atherosclerosis by lncRNA-MEG3 via Regulation of miR-26a/Smad1 Axis. Int Heart J.

[40] Rui X, Wu X, Rong Z (2023). Upgulation of lncRNA GASL1 inhibits atherosclerosis by regulating miR-106a/LKB1 axis. BMC Cardiovasc Disord.

[41] Chen H, Li X, Liu S (2017). MircroRNA-19a promotes vascular inflammation and foam cell formation by targeting HBP-1 in atherogenesis. Sci Rep.

[42] Dai Z, Liu X, Zeng H (2022). Long noncoding RNA HOTAIR facilitates pulmonary vascular endothelial cell apoptosis via DNMT1 mediated hypermethylation of Bcl-2 promoter in COPD. Respir Res.

[43] Lang YY, Xu XY, Liu YL (2022). Ghrelin Relieves Obesity-Induced Myocardial Injury by Regulating the Epigenetic Suppression of miR-196b Mediated by lncRNA HOTAIR. Obes Facts.

